# Developing and validating a discrete-event simulation model of multiple myeloma disease outcomes and treatment pathways using a national clinical registry

**DOI:** 10.1371/journal.pone.0308812

**Published:** 2024-08-27

**Authors:** Adam Irving, Dennis Petrie, Anthony Harris, Laura Fanning, Erica M. Wood, Elizabeth Moore, Cameron Wellard, Neil Waters, Kim Huynh, Bradley Augustson, Gordon Cook, Francesca Gay, Georgia McCaughan, Peter Mollee, Andrew Spencer, Zoe K. McQuilten

**Affiliations:** 1 Centre for Health Economics, Monash Business School, Monash University, Melbourne, Victoria, Australia; 2 Transfusion Research Unit, School of Public Health and Preventive Medicine, Monash University, Melbourne, Victoria, Australia; 3 Sir Charles Gairdner Hospital, Perth, Western Australia, Australia; 4 Leeds Institute of Clinical Trials Research, University of Leeds, Leeds, United Kingdom; 5 Division of Hematology, AOU Città della Salute e della Scienza di Torino, University of Turino, Torino, Italy; 6 Department of Haematology, St Vincent’s Hospital Sydney, Sydney, New South Wales, Australia; 7 Medicine & Health, University of New South Wales, Sydney, New South Wales, Australia; 8 Princess Alexandra Hospital, The University of Queensland, Brisbane, Queensland, Australia; 9 Australian Centre for Blood Diseases, Alfred Health–Monash University, Melbourne, Victoria, Australia; 10 Department of Malignant Haematology and Stem Cell Transplantation, Alfred Health, Melbourne, Victoria, Australia; Bursa Ali Osman Sonmez Oncology Hospital, TÜRKIYE

## Abstract

Multiple myeloma is a haematological malignancy typically characterised by neoplastic plasma cell infiltration of the bone marrow. Treatment for multiple myeloma consists of multi-line chemotherapy with or without autologous stem cell transplantation and has been rapidly evolving in recent years. However, clinical trials are unable to provide patients and clinicians with long-term prognostic information nor policymakers with the full body of evidence needed to perform economic evaluation of new therapies or make reimbursement decisions. To address these limitations of the available evidence, this study aimed to develop and validate the EpiMAP Myeloma model, a discrete-event simulation model of multiple myeloma disease outcomes and treatment pathways. Risk equations were estimated using the Australian and New Zealand Myeloma & Related Diseases Registry after multiple imputation of missing data. Risk equation coefficients were combined with multiple myeloma patients at diagnosis from the Registry to perform the simulation. The model was validated with 100 bootstraps of an out-of-sample prediction analysis using a 70/30 split of the 4,121 registry patients diagnosed between 2009 and 2023, resulting in 2,884 and 1,237 patients in the training and validation cohorts, respectively. For 90% of the 120 months in the 10-year post-diagnosis period, there was no significant difference in overall survival between the validation and simulated cohorts. These results highlight that the EpiMAP Myeloma model is robust at predicting multiple myeloma disease outcomes and treatment pathways in Australia & New Zealand. In the future, clinicians will be able to use the EpiMAP Myeloma model to provide personalised estimates of life expectancy to patients based on their specific characteristics, disease stage, and response to treatment. Policymakers will also be able to use the model to perform economic evaluation, to forecast the number of patients receiving treatment at different stages, and to determine the downstream impact of listing new, effective therapies.

## Introduction

Multiple myeloma (MM) is a haematological malignancy characterised by the proliferation of malignant plasma cells in the bone marrow. Manifestations of organ injury associated with MM commonly include calcium elevation (C), renal insufficiency (R), anaemia (A), and bone lesions (B): the CRAB criteria. The incidence of MM increases with age, with a mean age of 67 years at diagnosis [1]. Treatment for MM includes multi-line chemotherapy with or without autologous stem cell transplantation (ASCT). Despite recent advances in therapeutic options, MM remains incurable, with a median overall survival (OS) of approximately 6 years from diagnosis in Australia & New Zealand [1].

The treatment landscape for MM has evolved rapidly in recent years with development of new therapeutic agents, including second generation proteasome inhibitors, second generation immunomodulatory drugs, and anti-CD38 monoclonal antibodies [2]. New treatments are made available based on supportive evidence from clinical trials on their short-term efficacy on surrogate outcomes such as progression-free survival (PFS); [3, 4] however, because most patients are still alive at the end of follow-up, these clinical trials are unable to provide patients and clinicians with long-term prognostic information nor policymakers with the full body of evidence needed to perform economic evaluation or make reimbursement decisions. In addition, clinical trials often have restrictive inclusion criteria and thus do not fully represent real-world patients [5].

Predicting longer-term health outcomes is also important for informed decision-making by clinicians, patients, and their families [6]. In the MM literature, long-term prognostic information is available via the 3-level International Staging System (ISS) score developed by the International Myeloma Working Group (IMWG). The ISS was first published in 2005 and provided estimates of median OS for each level of the score [7]. The Revised ISS (R-ISS) was published in 2015, adding additional prognostic factors and updating the estimates of median OS [8]. While accurate at the time of publication, given the speed at which the treatment landscape for MM has evolved, after publication the data used to inform the ISS and R-ISS quickly began to lag behind current practice. This led to these scores losing their accuracy at predicting long-term health outcomes for newly diagnosed MM patients over time, as suggested by authors of a 2022 proposal for an updated, 4-level R2-ISS [9].

Epidemiological modelling using observational data from a real-world registry can provide more up-to-date predictions of long-term health outcomes and immediately incorporate the latest data as new therapies come to market [10]. Furthermore, epidemiological modelling is also useful in the health economics discipline to perform economic evaluation, to predict long-term resource use, and to inform healthcare decision-making as part of the health technology assessment (HTA) process [11]. The primary aim of this study was to develop and validate the EpiMAP Myeloma model, a discrete-event simulation model of MM disease outcomes and treatment pathways which will provide long-term prognostic information to patients and clinicians, and provide policymakers with the full body of evidence required to perform economic evaluation and make reimbursement decisions.

An important function of any model of a fatal disease is the means through which treatment conveys a survival benefit to patients. Cancer models typically employ PFS as a surrogate outcome for OS, whereby a treatment that demonstrates improved PFS will also improve OS [12]. A recent review found that between 2012 and 2022 the Pharmaceutical Benefits Advisory Committee, the committee that advises the Australian government on the reimbursement of new pharmaceuticals, made 19 decisions for treatments for MM based on a surrogate outcome because the data for the effect on OS was not yet mature [13]. However, defining progression in MM is often more complex than for solid tumours, relying on a clinicopathological assessment for diagnosis and staging [14]. Instead of imaging tests alone, the IMWG defines progressive disease a pre-defined increase in the quantity of serum and/or urinary protein biomarkers secreted by the MM cells [15]. In MM clinical trials, patients are followed-up intensively and progression is well captured as mandated by scheduled assessments. However, in real-world MM clinical practice, progression is often not documented by clinicians, as it may not always be a trigger for re-commencement of therapy, and hence may be poorly translated into registry-based data. The IMWG also provides standardised definitions for best clinical response (BCR) to MM treatment, a six-item ordinal scale ranging from Complete Response to Progressive Disease. BCR to treatment is generally better captured than progression in real-world data sources, such as clinical registries [15], and a recent systematic review found that BCR was a feasible surrogate outcome for OS [16]. The secondary aim of the study was to confirm that when modelling MM disease outcomes and treatment pathways, BCR to treatment could be used as a surrogate outcome for OS, instead of PFS.

## Materials and methods

We built the Epidemiological Modelling of Australian Patients with Myeloma model (EpiMAP Myeloma), a discrete-event simulation (DES) model using risk equations estimated from our national MM clinical registry–the Australian and New Zealand Myeloma & Related Diseases Registry (MRDR). DES modelling is often used to model resource constrained systems of capacity such as a hospital emergency department or operating theatre; however, DES can also be used to build disease models as an alternative framework to Markov cohort modelling [17]. As the name suggests, the unit of analysis in a Markov cohort model is a cohort of identical patients, generally assumed to have the mean set of characteristics for the population of interest. Markov cohort modelling is convenient because an analyst can utilise published summary data from clinical trials, such as hazard ratios, without the need for individual patient-level data. DES is usually performed at the individual patient level and therefore has significantly higher data requirements than cohort modelling. However, given sufficient high-quality data at the individual level, DES modelling is likely to be superior to Markov cohort modelling for several reasons. In a DES model, time is treated as a continuous variable rather than in cycles of pre-determined length, providing a closer approximation to the real-world experience and more natural predictions of the course of a disease. By design, DES models track the journey of individual patients through the disease, and can easily capture patient history, whereas Markov cohort models tend to be memoryless. Finally, as DES models are able to include more variables without aggregation, they utilise and are able to control for and explore the impact of individual patient characteristics on outcomes, such as OS or duration of therapy. A recent systematic review of MM modelling highlighted the need for a model such as the EpiMAP Myeloma model that is able to capture complex treatment strategies, noting that published models rarely followed an individual patient approach, mainly owing to the higher need for complex data assumptions [18].

### Data collection

The MRDR provided all individual patient data for this study. The MRDR was established in 2012, currently has 52 participating sites across Australia & New Zealand, and data on over 4,000 newly diagnosed MM patients [1, 19]. The MRDR captures detailed diagnostic and treatment characteristics for all enrolled patients and is linked to the National Death Index to ensure accurate recording of death [20–22]. The MRDR has ethics approval to operate as an opt-off clinical quality registry with discretion over how the collected data are utilised. The MRDR Steering Committee approved the request to utilise fully anonymised MRDR data to build the EpiMAP Myeloma model and standalone ethics approval including a waiver of consent for the modelling study was provided by Monash University (Project ID 26371).

All participants registered on the MRDR from inception to 16^th^ January 2024 were included in the study. All diagnosis, treatment, and outcome data were extracted for the analysis. Multiple imputation using chained equations was used to impute missing patient characteristic and BCR data, creating 10 complete, imputed datasets which were combined using Rubin’s rules to estimate our risk equations [23]. Additional details including the form and covariates included in each of the multiple imputation regressions are presented in S1 Table in the S1 File. Chemotherapy lines with no recorded end date that must have ended because the patient had since died or started a new line were dropped from the analysis to avoid skewing the data with very long, censored chemotherapy durations.

### Model design

**Fig 1:**
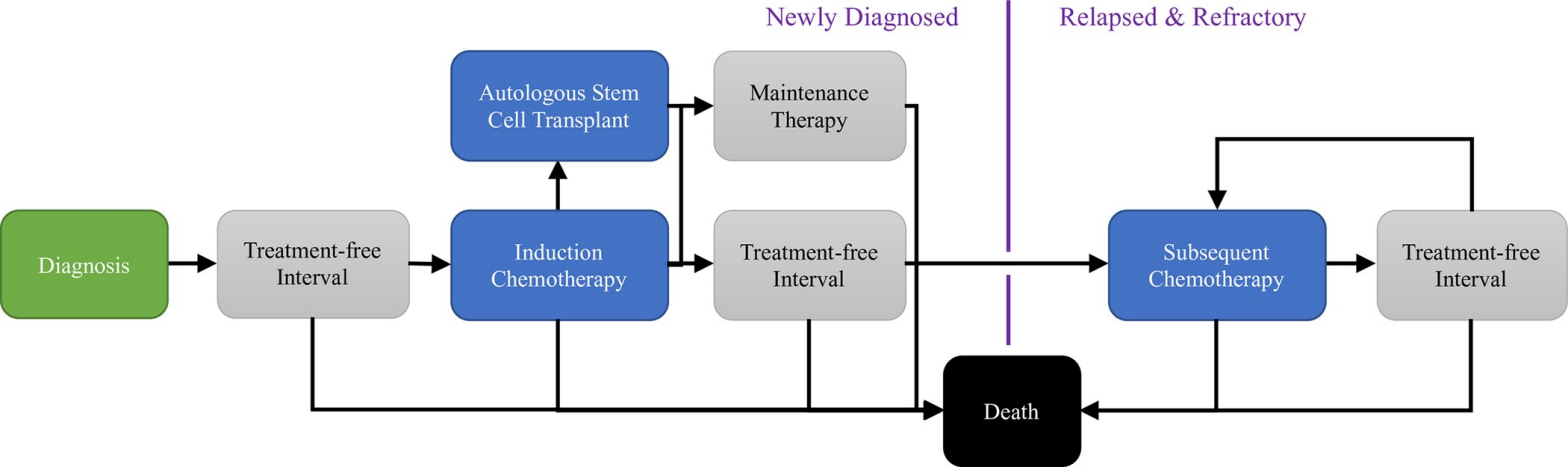
The EpiMAP myeloma model framework. Blue health states represent treatments where best clinical response is predicted, either to chemotherapy or autologous stem cell transplant. Grey health states represent treatment-free intervals or post-induction maintenance therapy.

Version 1.0 of the EpiMAP Myeloma model framework, presented in Fig 1, was designed in close collaboration with an advisory group of clinical MM experts to simplify the disease outcomes and treatment pathways but still reflect its key components. At MM diagnosis, patients are assigned four diagnostic characteristics—age, sex, Eastern Cooperative Oncology Group (ECOG) performance score and ISS score. ECOG is a 5-level score from 0 to 4; however, we combined the high-morbidity categories 2, 3 & 4 due to low numbers. We used ISS despite its supersession by the R-ISS because one key component of the R-ISS, the FISH cytogenetic risk score, is not routinely obtained in Australia & New Zealand and was missing for approximately half of the MRDR patients.

The EpiMAP Myeloma model (v1.0) considers up to nine LoTs and BCR is predicted after each treatment (chemotherapy or ASCT). The time between LoTs is defined as a treatment-free interval. Post-induction maintenance therapy is not expected to improve BCR; therefore, receiving maintenance therapy only increases the time between induction chemotherapy (LoT 1) and 1^st^ subsequent chemotherapy (LoT 2). For LoT 1 and LoT 2 we modelled those specific chemotherapy regimens that were used by at least 10% of MRDR patients who received that LoT, alongside ‘other’ chemotherapy. Given the wide variety of combinations of chemotherapeutic agents recorded in the MRDR, for LoT 3 onwards specific chemotherapy regimens were not modelled, with all patients instead receiving the survival benefit of the average chemotherapy regimen. The list of modelled chemotherapy regimens for LoT 1 and LoT 2 is presented in S2 Table in the S1 File. Additionally, beyond LoT 3 BCR was collapsed into a 3-item scale due to low numbers in some of the categories (1—Complete Response or Very Good Partial Response, 2—Partial Response or Minimal Response, and 3—Stable Disease or Progressive Disease). LoTs 6–9 were assumed to be equivalent, sharing the same OS coefficients and risk equations for chemotherapy duration, treatment-free interval, and BCR.

### Risk equations

We estimated several types of risk equations to simulate the disease, treatment, and outcome trajectory of MM patients through the EpiMAP Myeloma model framework. We used parametric survival analysis to model time-to-event data, including OS, chemotherapy duration, and treatment-free intervals. The parametric distribution for these outcomes were selected based on information criteria and visual inspection of the goodness-of-fit. Logit regression was used to model binary outcomes–planned ASCT, receipt of ASCT, and receipt of maintenance therapy. Multinomial logit regression was used to predict chemotherapy regimen and ordered logit for BCR to treatment. These risk equations were all estimated using the multiply imputed patient-level MRDR data. Covariates included in the risk equations were chosen based on availability of data and clinical plausibility via consultation with our advisory group of clinical MM experts. OS depended on patient characteristics including BCR to each LoT. BCR depended on patient characteristics, chemotherapy regimen, and BCR to the previous LoT. The complete list of covariates included in each risk equation is presented in S3 Table in the S1 File.

Given significant differences in outcomes, separate ASCT and non-ASCT regressions were performed for induction chemotherapy duration and LoT 1 to LoT 2 treatment-free interval. Furthermore, induction chemotherapy duration for patients with planned ASCT utilised three manual splines to capture rapidly changes hazard rates and patients whose BCR to induction chemotherapy was Stable Disease or Progressive Disease were ineligible for ASCT. In total the EpiMAP Myeloma model contains 30 risk equations. For transparency and reproducibility, a complete set of regression outputs from all 30 risk equations estimated on the full MRDR dataset is presented in S4 to S33 Tables in the S1 File.

### Simulation

Patient characteristics of the hypothetical MM patients to be simulated were based on the patients from the MRDR. For each decision point in the model framework (e.g., after induction chemotherapy does this patient receive an ASCT?), the coefficients from the respective risk equation were used to calculate the likelihood *p* of each patient experiencing the outcome. This was then compared to a random number *r* drawn from a uniform distribution between 0 and 1 to determine the outcome (e.g., if *p* < *r* this patient receives an ASCT). As a DES model, all time periods were modelled explicitly for each patient. OS was predicted at the start of each health state with random numbers drawn from a uniform distribution between 0 and each patient’s current position on their specific OS curve. When assessing the time to competing events such as death versus end of chemotherapy duration, the model selected whichever event happened first. An age limit was used to curtail patients whose survival was estimated beyond 100 years old. As the MRDR contained some missing chemotherapy end dates, chemotherapy duration and treatment-free intervals were curtailed at the maximum observed in the data. The simulation was performed in Stata 17 (StataCorp, College Station, Texas) using the in-built matrix language Mata on the MASSIVE high-performance computing infrastructure [24, 25].

### Analysis

To demonstrate the predictive power of the model we performed an out-of-sample prediction analysis. We randomly divided the MM patients in the MRDR into a 70% training cohort and a 30% validation cohort. The 70% training cohort was used to perform the multiple imputation and estimate the risk equations while the 30% validation cohort provided the diagnostic characteristics of the simulated patients and the holdout comparison group.

To characterise parameter uncertainty in the model, the training cohort was bootstrapped 100 times with separate multiple imputation, risk equations, and simulations performed for each bootstrapped sample. We compared the 95% confidence intervals of the OS Kaplan-Meier curves of validation and simulated cohorts. For each month in the 10-year post-diagnosis period we manually calculated the p-value under the null hypothesis that the two OS curves were equal. This was achieved by comparing the 100 bootstrap samples of two OS curve in each month. As an example, if OS in the validation cohort was greater than the simulation cohort in 50 out of the 100 bootstrap samples the p-value for that month would have been (50/100*2) = 1. If OS in the validation cohort was greater than the simulation cohort in 5 out of the 100 bootstrap samples then the p-value for the month would have been (5/100*2) = 0.1.

Finally, to demonstrate that BCR to treatment is a feasible surrogate outcome for OS, we broke down OS in the validation and simulated cohorts by BCR to LoT 1 / ASCT (LoT 1 for non-ASCT patients, ASCT for ASCT patients). To be appropriate in this context, there needs to be a relationship between BCR and OS such that patients who experienced improved BCR to treatment also experienced improved OS. Ideally, OS would be monotonically increasing from the worst BCR category to the best and no two BCR categories would have a similar expected OS.

### Data availability

The MRDR data are property of Monash University and not publicly available due to the sensitive patient information. De-identified data are available for research purposes upon successful application to the MRDR Steering Committee for researchers and projects that meet the criteria for access to confidential data, more information can be found at https://www.mrdr.net.au/. The Stata code for v1.0 of the EpiMAP Myeloma model used to perform the simulation has been made open source using the General Public License version 3 (GPLv3). Risk equation coefficients for the EpiMAP Myeloma model are presented in the Supplementary Information files and available on the online repository for use with the simulation code. The online repository can be found at https://github.com/adam-irving/EpiMAP-Myeloma/.

## Results

There were 4,121 MM patients included, 2,884 (70%) and 1,237 (30%) of whom were randomly allocated to the training and validation cohorts, respectively. The characteristics of these patients at diagnosis, before the imputation of missing values, are presented Table 1.

**Table 1:** Patient characteristics at diagnosis.

	Training	Validation
	n = 2,884	n = 1,237
Age (mean ± SD)	67.3 ± 11.6	67.8 ± 11.3
Male (%)	1,736 (60.4%)	758 (61.4%)
ECOG (%)		
Missing	832 (28.9%)	363 (29.4%)
0	880 (30.5%)	353 (28.5%)
1	757 (26.3%)	347 (28.1%)
2, 3 or 4	415 (14.4%)	174 (14.1%)
ISS (%)		
Missing	757 (26.3%)	344 (27.8%)
1	624 (21.6%)	236 (19.1%)
2	886 (30.7%)	379 (30.6%)
3	617 (21.4%)	278 (22.5%)

ECOG–Eastern Cooperative Oncology Group; ISS–International Staging System; SD–standard deviation

Patient characteristics at diagnosis with significant missing data in the registry were ECOG (29.6%) and ISS (26.8%). These were imputed with other diagnostic, comorbidity, and laboratory data. BCR to chemotherapy and ASCT had 29.0% and 26.6% missing data, respectively, which were imputed using changes in paraprotein, lambda light chain, and kappa light chain levels. Survival analysis results using the training cohort indicated that the optimal distribution in terms of goodness-of-fit as judged by information criteria and visual inspection were the Gompertz distribution for the OS equation and the Weibull distribution for the chemotherapy duration and treatment-free interval equations. Both of these distributions are commonly used in survival analysis for cancer modelling, the Gompertz distribution assumes that the hazard increases exponentially with time and the Weibull distribution assumes that the hazard increases at a constant rate over time.

The 100 bootstraps of the multiply imputed datasets of the validation cohort were used to define the diagnostic characteristics of the simulated patients. For each bootstrap, 10,000 patients were run through the EpiMAP Myeloma model from diagnosis to death over up to nine LoTs.

**Fig 2:**
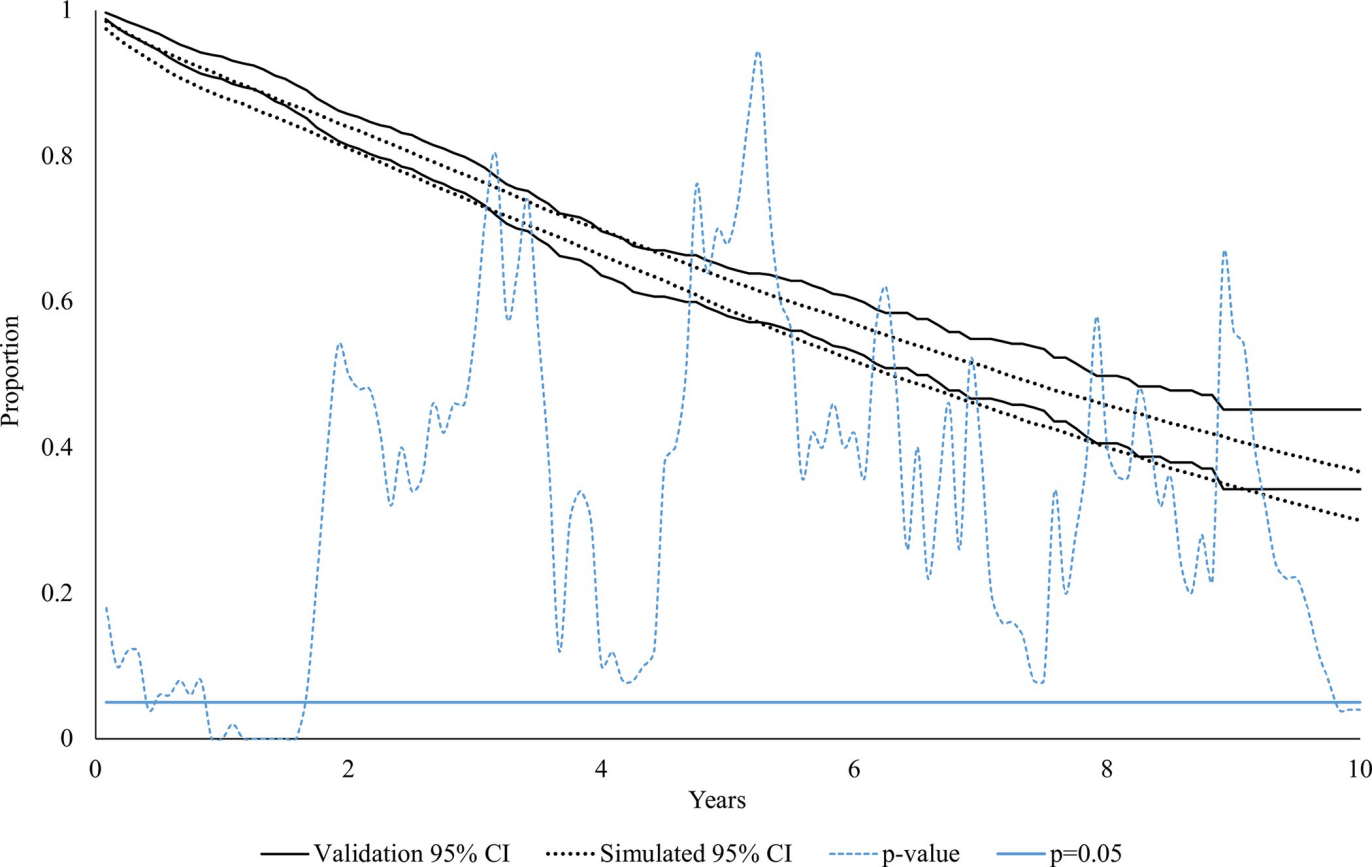
95% confidence intervals of the Kaplan-Meier OS curves for the validation and simulated cohorts. The dashed light blue line represents the monthly p-value evaluating the significance of the difference between the confidence intervals.

Fig 2 presents the 95% confidence intervals of the Kaplan-Meier OS curves for the validation and simulated cohorts and the corresponding monthly p-value evaluating statistical significance between the confidence intervals. There is good visual alignment between the two sets of confidence intervals, formally confirmed by the line representing monthly p-values. The p-value only drops below 0.05 in year 1, where the simulation briefly underestimates OS, and at the end of year 9, when there are very few patients alive in the validation cohort. For 90% of the 120 months in the 10-year post-diagnosis period, there is no significant difference in OS between the validation and simulated cohorts.

To demonstrate the validity of using BCR as a surrogate outcome for OS, Table 2 presents OS by BCR to LoT 1 / ASCT (LoT 1 for non-ASCT patients, ASCT for ASCT patients) for the validation and simulated cohorts.

**Table 2:** Overall survival by BCR to LoT 1 / ASCT.

Best Clinical Response to LoT 1 / ASCT	Validation cohort	Simulated cohort
	Median (25% - 75%)	Median (25% - 75%)
Complete Response	NR (4.9 –NR)	11.4 (6.5–16.3)
Very Good Partial Response	8.2 (4.0 –NR)	8.7 (4.7–13.5)
Partial Response	6.4 (2.9 –NR)	7.2 (3.9–11.9)
Minimal Response	6.7 (3.0 –NR)	6.5 (3.4–10.9)
Stable Disease	3.6 (1.5 –NR)	4.1 (2.1–7.1)
Progressive Disease	2.9 (0.8 –NR)	4.1 (2.2–7.2)

ASCT = autologous stem cell transplant; BCR = best clinical response; LoT = line of therapy; NR = not reached

Patients in the validation cohort had not reached the 75^th^ percentile of the OS distribution for all BCR categories and not reached the median for those who experienced Complete Response. In the validation cohort there is increasing OS with improved BCR; however, this relationship is not monotonically increasing as patients who experienced Minimal Response had marginally improved OS compared with Partial Response. Comparing the validation and simulated cohort, both the median and 25^th^ percentile of the OS distribution were most similar for the BCR categories in the middle of the scale, and most disparate for Complete Response and Progressive Disease at the ends of the scale.

**Fig 3:**
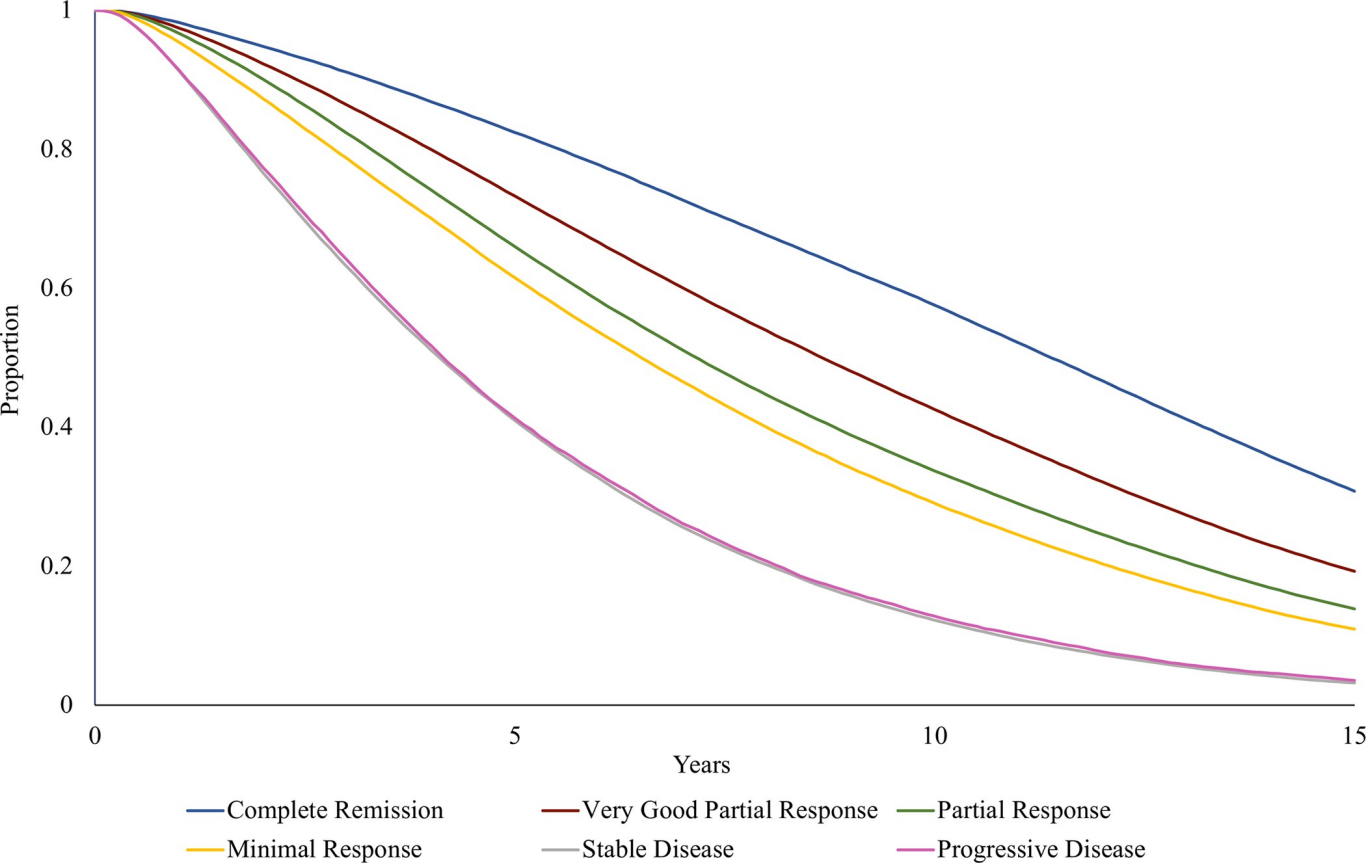
Overall survival curves by BCR to LoT 1 / ASCT for the simulated cohort.

Fig 3 presents the first 15 years of OS curves for the simulated cohort by BCR to LoT 1 / ASCT. There is no difference in OS between Stable Disease and Progressive Disease, and only a small difference between Partial Response and Minimal Response. This implies that when modelling OS in MM using BCR to treatment, there is little statistical power to distinguish a patient who experiences Stable Disease versus Progressive Disease or who experiences Partial Response versus Minimal Response to LoT 1 / ASCT.

## Discussion

The primary aim of this study was to develop and validate the EpiMAP Myeloma model, a discrete-event simulation model of MM disease outcomes and treatment pathways. To validate the model, we employed an out-of-sample prediction analysis, encompassing a 70% training cohort for conducting the multiple imputation and estimating the risk equations, and a 30% validation cohort defining the diagnostic characteristics of the simulated patients and the holdout comparison group. The results of the out-of-sample prediction analysis revealed that for 90% of the 120 months in the 10-year post-diagnosis period, there was no significant difference in OS between the validation and simulated cohorts. This finding underscores the robustness of the EpiMAP Myeloma model in accurately forecasting the OS outcomes of the validation cohort given the simulated results were generated independently of these patients. Additionally, this confirms the appropriateness of the internal design of the EpiMAP Myeloma model including the transitions between health states and the specification of the risk equations.

As reliable progression data were not available from the MRDR, the secondary aim of this study was to demonstrate that BCR to treatment, could be used as a surrogate outcome for OS, instead of PFS. This relationship could not be assumed, even given the results of the out-of-sample prediction analysis as the model could still accurately predict OS on average even if there were no differences in OS by BCR. Overall, we found a pattern of response that provides good evidence of the predictive power of differences in BCR to explain differences in OS; however, there are also clear statistical limitations. When considering BCR to LoT 1 / ASCT, the relationship between BCR and OS was not monotonically increasing in the validation cohort, and the model predicted similar OS outcomes for a patient who experienced Minimal Response versus Partial Response or who experienced Stable Disease versus Progressive Disease. BCR is a measure of depth of response which, while is clearly somewhat predictive of OS, is unlikely to capture the full dynamics of response to treatment. Primarily, BCR does not speak to the durability of response, which is also highly likely to influence PFS and OS. Altogether, BCR appears to be a reasonable, but not perfect, surrogate outcome for OS. As such, the current version of the EpiMAP Myeloma model is able to simulate the impact of listing a new therapy on MM disease outcomes through the therapy’s ability to improve BCR to treatment. Nevertheless, there is likely to be room for improvement, future modelling work could further explore the relationship between BCR and PFS and assess the impact of modelling PFS directly in lieu of high-quality progression data, or the potential for including both BCR and PFS in order to measure the gains from including PFS as well.

Our study has several further limitations. Firstly, our analysis utilises data from Australia & New Zealand only, and may have limited generalisability to MM patients in other jurisdictions which employ alternative treatment strategies. Furthermore, several important variables had a non-trivial amount of missing data. Indeed, we were unable to use R-ISS because approximately half of our real-world patient population did not receive the necessary diagnostic tests to calculate this prognostic indicator. The remaining patient characteristics were imputed using multiple imputation, as were BCR to both chemotherapy and to ASCT. Multiple imputation can introduce bias if not handled appropriately. The MRDR captures a significant number of prognostic variables at diagnosis and treatment which we were able to include in our imputation regressions which should have minimised any potential bias from model misspecification. Multiple imputation also assumes that the missing data are missing at random, that is, unrelated to the observed, non-missing values. We found no evidence that the missing data were related to the patient, or the treatment received. MRDR data are manually entered by nurses at each hospital site who are not reimbursed for their time, we assume that the missing data is missing at random because it is due to the time poor nature of the hospital environment, rather than due to any observed or unobserved patient or treatment characteristics. Another limitation of the data was that the MRDR contains some missing end dates for lines of chemotherapy. Rather than make assumptions about how long chemotherapy was expected to last, we decided to drop chemotherapy lines that we knew must have ended because the patient had since died or started a new line of chemotherapy. Thankfully, this only occurred for approximately 300 of the 7,500 lines of chemotherapy in the MRDR. Finally, as a model is a simplification of the real-world, there were instances of treatments received by MM patients in the MRDR that were not included in the EpiMAP Myeloma model. These include patients who had multiple ASCTs, or who had an ASCT or maintenance therapy after subsequent chemotherapy rather than induction chemotherapy. We chose not to model these possibilities either because the number of patients were small, or they did not appear to have a significant impact on disease outcomes.

As the treatment landscape for MM evolves there is a need for more complex modelling such as the EpiMAP Myeloma model to predict patient-level, long-term outcomes, as highlighted by a recent systematic review of the MM modelling literature [18]. Common sources of short-term follow-up data such as clinical trials are unable to provide clinicians with long-term prognostic information nor policymakers with the full body of evidence needed to perform economic evaluation or to make reimbursement decisions. Furthermore, recently published research in both the US and Australia has found that the OS benefit of new therapies reported in clinical trials is rarely replicated when the treatment is approved and delivered in routine clinical practice [26, 27]. The authors highlight the differences in patient characteristics and treatment adherence between clinical trials and routine clinical practice. Until recently, despite the widely acknowledged shortcoming in the external validity of clinical trials, policymakers have not had ready access to alternative estimates of real-world long-term OS to inform their decisions. As registries such as the MRDR mature and accompanying models such as EpiMAP Myeloma model are developed, we believe policymakers will be able to incorporate long-term OS estimates derived directly from data on the real-world patients for whom they are making decisions.

The EpiMAP Myeloma model sits at the intersection between decision-analytic and epidemiological modelling. Epidemiological modelling is commonly performed in cancer research and is useful for prediction and forecasting the number of patients at different stages of a disease over time. Compartmental models are an established epidemiological technique often used to model infectious diseases in a dynamic systems approach but have also been used in cancer. A recent US epidemiological study in MM used a compartmental model to estimate the number of patients receiving treatment by LoT [10]. This type of model is effective for understanding trends in incidence and prevalence which can be used by healthcare planners to inform resource allocation decisions. However, we argue that DES models are an improvement upon compartmental models, as they allow for a more detailed representation of disease outcomes and treatment pathways, accommodate individual patient characteristics, and utilise time-dependent events. These same attributes imbue DES models with a similar advantage over Markov cohort models in the field of decision-analytic modelling. Beyond the model design and structure, another strength of our study includes the large, multicentre, prospectively collected dataset of real-world patients that enabled the DES modelling.

Modelling is a powerful tool that can be used to perform deeper enquiries of observational data than those which are possible with the data alone. The validated EpiMAP Myeloma model could now be used by government agencies as a reference model in HTA processes to provide estimates of the future number of MM patients who will receive treatment at different LoTs, or, once combined with clinical trial, quality-of-life, and resource use data, the cost-effectiveness of new or existing MM treatments based on the real-world Australia & New Zealand MM population. Alternatively, the EpiMAP Myeloma model could explore the expected value of a potential change in routine clinical practice, such as performing ASCT on older MM patients. Further research currently underway will provide more validation of the model in order to demonstrate its utility to stakeholders and promote its adoption by clinicians and policymakers [28]. Afterwards, we plan to make an interactive version of the EpiMAP Myeloma model available to clinicians through the MRDR website so that they can provide their patients with personalised estimates of life expectancy based on their specific characteristics, disease stage, and response to treatment. We would also like to validate our model against international data to determine whether the model is generalisable to jurisdictions which employ alternative treatment strategies. The EpiMAP Myeloma model will become a living model alongside the MRDR, future analyses run using the model will be able to utilise the most up-to-date registry data, improving the predictive accuracy of the risk equations and able to reflect changes in MM treatment patterns and outcomes as they occur.

To conclude, the results of our analysis provide confirmation of the predictive capacity of the EpiMAP Myeloma model (v1.0). The design and structure of the model is appropriate for simulating MM disease outcomes and treatment pathways with risk equations estimated using real-world registry data. We also demonstrated the validity of using BCR as a surrogate outcome for OS, instead of PFS.

## Supporting information

S1 File Supporting information file. (DOCX)
