# Supplementary material for: Developing and validating a discrete-event simulation model of multiple myeloma disease outcomes and treatment pathways using a national clinical registry
Source: PLoS One. 2024 Aug 27;19(8):e0308812. doi: 10.1371/journal.pone.0308812 (PMC11349175; doi:10.1371/journal.pone.0308812)
Supplement: S1 File — (DOCX) [file pone.0308812.s001.docx]

# SUPPORTING INFORMATION

S1 Table: Multiple Imputation Equations

| Equation^†^ | Imputed covariates | Form |
| --- | --- | --- |
| Diagnosis | Albumin, Alkaline Phosphatase, Bone Marrow Plasma Cells, Lactate Dehydrogenase, Haemoglobin, Neutrophil Count, Beta 2 Microglobulin, Serum Calcium, Serum Creatine, eGFR, EQ-5D-3L | Linear |
|  | FISH Risk, Extra Medullary Disease, Cardiac Disease, Pulmonary Disease, Diabetes, Liver Disease, Peripheral Neuropathy & Other Malignancy | Logit |
|  | cECOG­­ | Ordered logit |
| BCR to Chemotherapy | Change in paraprotein, change in lambda light chain, change in kappa light chain | Linear |
|  | BCR | Ordered logit |
| BCR to ASCT | Change in paraprotein, change in lambda light chain, change in kappa light chain | Linear |
|  | BCR | Ordered logit |

^†^Age was a regular covariate in all equations and LoT was a regular covariate in the Chemotherapy BCR equation

ASCT – autologous stem cell transplant; BCR – best clinical response, cECOG – collapsed Eastern Cooperative Oncology Group; eGFR –estimated glomerular filtration rate; EQ-5D-3L – EuroQol 5 Dimensions 3 Levels; FISH - fluorescence in situ hybridization; LoT – line of therapy

S2 Table: Modelled Chemotherapy Regimens

| LoT | Regimen | MRDR Market Share |
| --- | --- | --- |
| 1 | Bortezomib, cyclophosphamide & dexamethasone (VCd) | 58% |
|  | Bortezomib, lenalidomide & dexamethasone (VRd) | 15% |
|  | Other | 26% |
| 2 | Lenalidomide & dexamethasone (Rd) | 16% |
|  | Daratumumab, bortezomib & dexamethasone (DVd) | 11% |
|  | Other | 73% |

MRDR – Myeloma and Related Diseases Registry

S3 Table: Risk Equations - Summary

| Equation | Form | Covariates |
| --- | --- | --- |
| Overall Survival | Parametric survival – Gompertz | Age, Age^2^, Male, cECOG, ISS, BCR interacted with LoT |
| Diagnosis to Treatment Interval | Parametric survival – Weibull | Age, Male, cECOG, ISS, Planned ASCT |
| Treatment-free Interval | Parametric survival – Weibull | Age, Male, cECOG, ISS, BCR |
| Chemotherapy Regimen | Multinomial logit | Age, Male, cECOG, ISS, Previous LoT Regimens |
| Chemotherapy Duration | Parametric survival – Weibull | Age, Male, cECOG, ISS, Regimen |
| Planned ASCT | Logit | Age, Male, cECOG, ISS, Age ≥ 70, Age ≥75, Comorbidities^†^ |
| Receipt of ASCT | Logit | Age, Age^2^, Male, cECOG, ISS, Age ≥ 70, Age ≥ 75, Comorbidities^†^, BCR |
| Receipt of MNT | Logit | Age, Male, cECOG, ISS, ASCT, Regimen, BCR |
| BCR to Chemotherapy | Ordered logit | Age, Male, cECOG, ISS, Regimen, BCR |
| BCR to ASCT | Ordered logit | Age, Male, cECOG, ISS, BCR |

^†^Comorbidities include cardiac disease, pulmonary disease, diabetes, liver disease, peripheral neuropathy, and other malignancy

ASCT – autologous stem cell transplant; BCR – best clinical response, cECOG – collapsed Eastern Cooperative Oncology Group; ISS – international staging system; LoT – line of therapy; MNT – maintenance therapy

S4 Table: Risk Equation – Overall Survival

| Covariate | Coefficient | Std. Err. | Covariate | Coefficient | Std. Err. |
| --- | --- | --- | --- | --- | --- |
| Age | -0.065 | 0.018 | L2 – CR | -0.699 | 0.213 |
| Age^2^ | 0.001 | 0.000 | L2 – VG | -0.761 | 0.176 |
| Male | 0.042 | 0.055 | L2 – PR | -0.156 | 0.133 |
| cECOG |  |  | L2 – MR | -0.098 | 0.224 |
| 1 | 0.262 | 0.074 | L2 – SD | -0.283 | 0.197 |
| 2, 3 & 4 | 0.610 | 0.078 | L2 – PD | 0.965 | 0.211 |
| ISS |  |  | L3 – CR/VG | -0.106 | 0.189 |
| 2 | 0.321 | 0.085 | L3 – PR/MR | 0.128 | 0.150 |
| 3 | 0.747 | 0.089 | L3 – SD/PD | 1.005 | 0.141 |
| LoT#BCR |  |  | L4 – CR/VG | 0.373 | 0.242 |
| L1 – CR | -1.101 | 0.204 | L4 – PR/MR | 0.874 | 0.165 |
| L1 – VG | -0.833 | 0.149 | L4 – SD/PD | 1.525 | 0.156 |
| L1 – PR | -0.499 | 0.134 | L5 – CR/VG | 0.904 | 0.298 |
| L1 – MR | 0.129 | 0.198 | L5 – PR/MR | 1.333 | 0.210 |
| L1 – SD | 0.171 | 0.116 | L5 – SD/PD | 1.592 | 0.185 |
| L1 – PD | 0.660 | 0.294 | L6+ – CR/VG | 0.189 | 0.464 |
| ASCT – CR | -1.101 | 0.207 | L6+ – PR/MR | 1.155 | 0.226 |
| ASCT – VG | -1.005 | 0.196 | L6+ – SD/PD | 2.464 | 0.191 |
| ASCT – PR | -1.108 | 0.239 | constant | -1.922 | 0.627 |
| ASCT – MR | -0.377 | 0.249 | gamma | 0.005 | 0.015 |

ASCT – autologous stem cell transplant; BCR – best clinical response; cECOG – collapsed Eastern Cooperative Oncology Group; CR – Complete Response; ISS – international staging system; MR – Minimal Response; PR – Partial Response; PD – Progressive Disease; SD – Stable Disease; VG – Very Good Partial Response

S5 Table: Risk Equation – Planned ASCT

| Covariate | Coefficient | Std. Err. | Covariate | Coefficient | | Std. Err. |
| --- | --- | --- | --- | --- | --- | --- |
| Age | -0.034 | 0.006 | Age ≥ 75 | -3.477 | | 0.463 |
| Male | 0.109 | 0.084 | Cardiac disease | -0.588 | | 0.159 |
| cECOG |  |  | Pulmonary disease | -0.739 | | 0.236 |
| 1 | -0.135 | 0.103 | Diabetes | -0.502 | | 0.138 |
| 2, 3 & 4 | -0.610 | 0.133 | Liver disease | -0.576 | | 0.350 |
| ISS |  |  | Peripheral neuropathy | -0.544 | | 0.287 |
| 2 | -0.384 | 0.105 | Other malignancy | 0.039 | | 0.154 |
| 3 | -0.636 | 0.126 | constant | 3.019 | | 0.359 |
| Age ≥ 70 | -1.457 | 0.134 |  |  |  | |

cECOG – collapsed Eastern Cooperative Oncology Group; ISS – international staging system

S6 Table: Risk Equation – Diagnosis Treatment-free Interval

| Covariate | Coefficient | Std. Err. | Covariate | Coefficient | Std. Err. |
| --- | --- | --- | --- | --- | --- |
| Age | 0.005 | 0.002 | 2 | 0.193 | 0.050 |
| Male | 0.004 | 0.034 | 3 | 0.378 | 0.064 |
| cECOG |  |  | ASCT Planned | 0.834 | 0.041 |
| 1 | 0.236 | 0.055 | constant | -3.230 | 0.136 |
| 2, 3 & 4 | 0.343 | 0.060 | ln(shape) | -0.572 | 0.012 |
| ISS |  |  |  |  |  |

ASCT – autologous stem cell transplant; cECOG – collapsed Eastern Cooperative Oncology Group; ISS – international staging system

S7 Table: Risk Equation – LoT 1 Chemotherapy Regimen

| Covariate | Coefficient | Std. Err. | Covariate | Coefficient | Std. Err. | |
| --- | --- | --- | --- | --- | --- | --- |
| **VCd** | | | **VRd** | | |  |
| Age | -0.080 | 0.004 | Age | -0.080 | 0.005 | |
| Male | 0.111 | 0.083 | Male | 0.092 | 0.111 | |
| cECOG |  |  | cECOG |  |  | |
| 1 | -0.108 | 0.113 | 1 | -0.333 | 0.153 | |
| 2, 3 & 4 | -0.438 | 0.123 | 2, 3 & 4 | -0.735 | 0.174 | |
| ISS |  |  | ISS |  |  | |
| 2 | 0.094 | 0.114 | 2 | -0.158 | 0.146 | |
| 3 | -0.003 | 0.121 | 3 | -0.487 | 0.155 | |
| constant | 6.397 | 0.313 | constant | 5.502 | 0.383 | |

cECOG – collapsed Eastern Cooperative Oncology Group; ISS – international staging system; VCd - Bortezomib, cyclophosphamide & dexamethasone; VRd - Bortezomib, lenalidomide & dexamethasone

S8 Table: Risk Equation – LoT 1 Chemotherapy Duration ASCT Planned, Spline 1 (0-60 days)

| Covariate | Coefficient | Std. Err. | Covariate | Coefficient | Std. Err. |
| --- | --- | --- | --- | --- | --- |
| Age | -0.027 | 0.014 | 3 | -0.250 | 0.403 |
| Male | -0.250 | 0.265 | Regimen |  |  |
| cECOG |  |  | VCd | -0.594 | 0.341 |
| 1 | 0.346 | 0.366 | VRd | -1.103 | 0.485 |
| 2, 3 & 4 | 0.431 | 0.430 | constant | -11.699 | 1.655 |
| ISS |  |  | ln(shape) | 0.943 | 0.131 |
| 2 | 0.306 | 0.308 |  |  |  |

cECOG – collapsed Eastern Cooperative Oncology Group; ISS – international staging system; VCd - Bortezomib, cyclophosphamide & dexamethasone; VRd - Bortezomib, lenalidomide & dexamethasone

S9 Table: Risk Equation – LoT 1 Chemotherapy Duration ASCT Planned, Spline 2 (60-100 days)

| Covariate | Coefficient | Std. Err. | Covariate | Coefficient | Std. Err. |
| --- | --- | --- | --- | --- | --- |
| Age | -0.014 | 0.004 | 3 | 0.139 | 0.096 |
| Male | 0.003 | 0.072 | Regimen |  |  |
| cECOG |  |  | VCd | 0.355 | 0.123 |
| 1 | -0.114 | 0.088 | VRd | 0.041 | 0.142 |
| 2, 3 & 4 | -0.195 | 0.129 | constant | -18.163 | 0.979 |
| ISS |  |  | ln(shape) | 1.36 | 0.05 |
| 2 | -0.053 | 0.086 |  |  |  |

cECOG – collapsed Eastern Cooperative Oncology Group; ISS – international staging system; VCd - Bortezomib, cyclophosphamide & dexamethasone; VRd - Bortezomib, lenalidomide & dexamethasone

S10 Table: Risk Equation – LoT 1 Chemotherapy Duration ASCT Planned, Spline 3 (≥100 days)

| Covariate | Coefficient | Std. Err. | Covariate | Coefficient | Std. Err. |
| --- | --- | --- | --- | --- | --- |
| Age | 0.001 | 0.005 | 3 | -0.257 | 0.129 |
| Male | -0.046 | 0.087 | Regimen |  |  |
| cECOG |  |  | VCd | -0.046 | 0.130 |
| 1 | -0.059 | 0.096 | VRd | 0.11 | 0.15 |
| 2, 3 & 4 | -0.244 | 0.142 | constant | -2.812 | 1.033 |
| ISS |  |  | ln(shape) | -0.185 | 0.189 |
| 2 | -0.063 | 0.098 |  |  |  |

cECOG – collapsed Eastern Cooperative Oncology Group; ISS – international staging system; VCd - Bortezomib, cyclophosphamide & dexamethasone; VRd - Bortezomib, lenalidomide & dexamethasone

S11 Table: Risk Equation – LoT 1 Chemotherapy Duration No ASCT Planned

| Covariate | Coefficient | Std. Err. | Covariate | Coefficient | Std. Err. |
| --- | --- | --- | --- | --- | --- |
| Age | -0.015 | 0.002 | 3 | 0.012 | 0.077 |
| Male | -0.145 | 0.049 | Regimen |  |  |
| cECOG |  |  | VCd | 0.202 | 0.055 |
| 1 | -0.021 | 0.062 | VRd | -0.273 | 0.087 |
| 2, 3 & 4 | 0.029 | 0.069 | constant | -6.060 | 0.235 |
| ISS |  |  | ln(shape) | 0.268 | 0.019 |
| 2 | 0.055 | 0.077 |  |  |  |

cECOG – collapsed Eastern Cooperative Oncology Group; ISS – international staging system; VCd - Bortezomib, cyclophosphamide & dexamethasone; VRd - Bortezomib, lenalidomide & dexamethasone

S12 Table: Risk Equation – LoT 1 Best Clinical Response

| Covariate | Coefficient | Std. Err. | Covariate | Coefficient | Std. Err. |
| --- | --- | --- | --- | --- | --- |
| Age | 0.009 | 0.003 | Regimen | -0.271 | 0.075 |
| Male | 0.114 | 0.060 | VCd | -0.643 | 0.102 |
| cECOG |  |  | VRd | -1.635 | 0.222 |
| 1 | -0.026 | 0.074 | cut1 | -0.129 | 0.220 |
| 2, 3 & 4 | 0.216 | 0.099 | cut2 | 1.233 | 0.220 |
| ISS |  |  | cut3 | 1.597 | 0.221 |
| 2 | 0.00 | 0.08 | cut4 | 4.322 | 0.246 |
| 3 | -0.007 | 0.088 | cut5 | -0.271 | 0.075 |

cECOG – collapsed Eastern Cooperative Oncology Group; ISS – international staging system; VCd - Bortezomib, cyclophosphamide & dexamethasone; VRd - Bortezomib, lenalidomide & dexamethasone

S13 Table: Risk Equation – Receipt of ASCT

| Covariate | Coefficient | Std. Err. | Covariate | Coefficient | Std. Err. |
| --- | --- | --- | --- | --- | --- |
| Age | 0.278 | 0.076 | MR | -2.354 | 0.270 |
| Age^2^ | -0.003 | 0.001 | SD | -0.458 | 0.200 |
| Male | 0.330 | 0.108 | Age > 70 | -1.014 | 0.201 |
| cECOG |  |  | Age > 75 | -2.596 | 0.542 |
| 1 | -0.379 | 0.127 | Cardiac disease | -0.861 | 0.190 |
| 2, 3 & 4 | -0.898 | 0.159 | Pulmonary disease | -0.990 | 0.282 |
| ISS |  |  | Diabetes | -0.743 | 0.166 |
| 2 | -0.447 | 0.141 | Liver disease | -0.725 | 0.379 |
| 3 | -0.699 | 0.159 | Peripheral neuropathy | -0.422 | 0.350 |
| BCR |  |  | Other malignancy | -0.213 | 0.180 |
| VG | -0.230 | 0.181 | constant | -4.294 | 2.114 |
| PR | -0.463 | 0.175 |  |  |  |

BCR – best clinical response; cECOG – collapsed Eastern Cooperative Oncology Group; ISS – international staging system; MR – Minimal Response; PR – Partial Response; SD – Stable Disease; VG – Very Good Partial Response

S14 Table: Risk Equation – ASCT Best Clinical Response

| Covariate | Coefficient | Std. Err. | Covariate | Coefficient | Std. Err. |
| --- | --- | --- | --- | --- | --- |
| Age | 0.009 | 0.006 | BCR | 2.507 | 0.226 |
| Male | 0.125 | 0.103 | VG | 3.956 | 0.233 |
| cECOG |  |  | PR | 4.936 | 0.364 |
| 1 | -0.138 | 0.124 | MR | 5.515 | 0.269 |
| 2, 3 & 4 | 0.127 | 0.189 | SD | 2.615 | 0.427 |
| ISS |  |  | cut1 | 4.659 | 0.437 |
| 2 | 0.122 | 0.123 | cut2 | 6.277 | 0.445 |
| 3 | 0.032 | 0.150 | cut3 | 2.507 | 0.226 |

BCR – best clinical response; cECOG – collapsed Eastern Cooperative Oncology Group; ISS – international staging system; MR – Minimal Response; PR – Partial Response; SD – Stable Disease; VG – Very Good Partial Response

S15 Table: Risk Equation – Receipt of Maintenance Therapy

| Covariate | Coefficient | Std. Err. | Covariate | Coefficient | Std. Err. |
| --- | --- | --- | --- | --- | --- |
| Age | -0.032 | 0.004 | VRd | 1.212 | 0.147 |
| Male | 0.153 | 0.086 | SCT | 1.522 | 0.099 |
| cECOG |  |  | BCR |  |  |
| 1 | -0.170 | 0.097 | VG | -0.137 | 0.143 |
| 2, 3 & 4 | -0.519 | 0.139 | PR | -0.124 | 0.139 |
| ISS |  |  | MR | 0.211 | 0.197 |
| 2 | -0.089 | 0.105 | SD | -0.634 | 0.161 |
| 3 | -0.341 | 0.122 | PD | -1.020 | 0.457 |
| Regimen |  |  | constant | 0.903 | 0.373 |
| VCd | 0.089 | 0.116 |  |  |  |

BCR – best clinical response; cECOG – collapsed Eastern Cooperative Oncology Group; ISS – international staging system; MR – Minimal Response; PR – Partial Response; PD – Progressive Disease; SD – Stable Disease; VCd - Bortezomib, cyclophosphamide & dexamethasone; VG – Very Good Partial Response; VRd - Bortezomib, lenalidomide & dexamethasone

S16 Table: Risk Equation – LoT 1 Treatment-free Interval – ASCT Patients

| Covariate | Coefficient | Std. Err. | Covariate | Coefficient | Std. Err. |
| --- | --- | --- | --- | --- | --- |
| Age | -0.002 | 0.005 | MNT | -0.665 | 0.083 |
| Male | 0.182 | 0.087 | BCR |  |  |
| cECOG |  |  | VG | 0.317 | 0.108 |
| 1 | 0.125 | 0.104 | PR | 0.418 | 0.117 |
| 2, 3 & 4 | 0.092 | 0.151 | MR | 0.619 | 0.136 |
| ISS |  |  | constant | -8.098 | 0.408 |
| 2 | 0.032 | 0.101 | ln(shape) | 0.038 | 0.035 |
| 3 | 0.153 | 0.123 |  |  |  |

BCR – best clinical response; cECOG – collapsed Eastern Cooperative Oncology Group; ISS – international staging system; MNT – Maintenance Therapy; MR – Minimal Response; PR – Partial Response; VG – Very Good Partial Response

S17 Table: Risk Equation – LoT 1 Treatment-free Interval – No ASCT Patients

| Covariate | Coefficient | Std. Err. | Covariate | Coefficient | Std. Err. |
| --- | --- | --- | --- | --- | --- |
| Age | -0.023 | 0.003 | BCR |  |  |
| Male | 0.065 | 0.059 | VG | 0.592 | 0.126 |
| cECOG |  |  | PR | 1.055 | 0.120 |
| 1 | 0.099 | 0.082 | MR | 1.680 | 0.135 |
| 2, 3 & 4 | -0.007 | 0.082 | SD | 1.024 | 0.134 |
| ISS |  |  | PD | 1.877 | 0.171 |
| 2 | 0.054 | 0.090 | constant | -2.238 | 0.240 |
| 3 | 0.170 | 0.089 | ln(shape) | -0.738 | 0.023 |
| MNT | -0.221 | 0.078 |  |  |  |

BCR – best clinical response; cECOG – collapsed Eastern Cooperative Oncology Group; ISS – international staging system; MNT – Maintenance Therapy; MR – Minimal Response; PR – Partial Response; PD – Progressive Disease; SD – Stable Disease; VG – Very Good Partial Response

S18 Table: Risk Equation – LoT 2 Chemotherapy Regimen

| Covariate | Coefficient | Std. Err. | Covariate | Coefficient | Std. Err. | |
| --- | --- | --- | --- | --- | --- | --- |
| **Rd** | | | **DVd** | | |  |
| Age | 0.036 | 0.007 | Age | 0.033 | 0.008 | |
| Male | 0.119 | 0.134 | Male | -0.139 | 0.149 | |
| cECOG |  |  | cECOG |  |  | |
| 1 | 0.105 | 0.171 | 1 | -0.018 | 0.209 | |
| 2, 3 & 4 | 0.191 | 0.211 | 2, 3 & 4 | -0.275 | 0.236 | |
| ISS |  |  | ISS |  |  | |
| 2 | -0.391 | 0.194 | 2 | 0.103 | 0.205 | |
| 3 | -0.166 | 0.177 | 3 | 0.151 | 0.205 | |
| LoT 1 Regimen |  |  | LoT 1 Regimen |  |  | |
| VCd | -0.013 | 0.158 | VCd | -0.514 | 0.180 | |
| VRd | -0.340 | 0.311 | VRd | 1.283 | 0.223 | |
| constant | -3.974 | 0.539 | constant | -3.922 | 0.602 | |

cECOG – collapsed Eastern Cooperative Oncology Group; ISS – international staging system; DVd – Daratumumab, bortezomib & dexamethasone; Rd – Lenalidomide & dexamethasone; VCd - Bortezomib, cyclophosphamide & dexamethasone; VRd - Bortezomib, lenalidomide & dexamethasone

S19 Table: Risk Equation – LoT 2 Chemotherapy Duration

| Covariate | Coefficient | Std. Err. | Covariate | Coefficient | Std. Err. |
| --- | --- | --- | --- | --- | --- |
| Age | 0.000 | 0.003 | DVd | -0.180 | 0.094 |
| Male | -0.082 | 0.057 | BCR |  |  |
| cECOG |  |  | VG | 0.148 | 0.095 |
| 1 | 0.100 | 0.071 | PR | 0.080 | 0.094 |
| 2, 3 & 4 | 0.138 | 0.087 | MR | 0.082 | 0.107 |
| ISS |  |  | SD | 0.172 | 0.118 |
| 2 | 0.128 | 0.083 | PD | 0.205 | 0.167 |
| 3 | 0.235 | 0.081 | constant | -4.801 | 0.223 |
| Regimen |  |  | ln(shape) | -0.252 | 0.022 |
| Rd | -0.587 | 0.080 |  |  |  |

BCR – best clinical response; cECOG – collapsed Eastern Cooperative Oncology Group; ISS – international staging system; MR – Minimal Response; PR – Partial Response; PD – Progressive Disease; SD – Stable Disease; VG – Very Good Partial Response

S20 Table: Risk Equation – LoT 2 Best Clinical Response

| Covariate | Coefficient | Std. Err. | Covariate | Coefficient | Std. Err. |
| --- | --- | --- | --- | --- | --- |
| Age | 0.000 | 0.004 | MR | 1.620 | 0.227 |
| Male | 0.013 | 0.086 | SD | 1.942 | 0.220 |
| cECOG |  |  | PD | 2.258 | 0.310 |
| 1 | 0.133 | 0.104 | BCR SCT |  |  |
| 2, 3 & 4 | 0.013 | 0.126 | CR | -1.305 | 0.202 |
| ISS |  |  | VG | -0.687 | 0.147 |
| 2 | 0.083 | 0.117 | PR | -0.266 | 0.164 |
| 3 | 0.234 | 0.122 | MR | 0.492 | 0.207 |
| Regimen |  |  | cut1 | -0.879 | 0.373 |
| Rd | 0.207 | 0.115 | cut2 | 0.621 | 0.373 |
| DVd | 0.502 | 0.129 | cut3 | 2.187 | 0.376 |
| BCR LoT 1 |  |  | cut4 | 2.717 | 0.377 |
| VG | 0.590 | 0.203 | cut5 | 3.917 | 0.383 |
| PR | 1.251 | 0.200 |  |  |  |

BCR – best clinical response; cECOG – collapsed Eastern Cooperative Oncology Group; DVd – Daratumumab, bortezomib & dexamethasone; ISS – international staging system; MR – Minimal Response; PR – Partial Response; PD – Progressive Disease; Rd – Lenalidomide & dexamethasone; SD – Stable Disease; VG – Very Good Partial Response

S21 Table: Risk Equation – LoT 2 Treatment-free Interval

| Covariate | Coefficient | Std. Err. | Covariate | Coefficient | Std. Err. |
| --- | --- | --- | --- | --- | --- |
| Age | 0.001 | 0.003 | BCR |  |  |
| Male | -0.048 | 0.068 | VG | 0.294 | 0.133 |
| cECOG |  |  | PR | 0.525 | 0.125 |
| 1 | 0.183 | 0.081 | MR | 1.041 | 0.147 |
| 2, 3 & 4 | 0.197 | 0.096 | SD | 0.902 | 0.143 |
| ISS |  |  | PD | 1.807 | 0.148 |
| 2 | 0.038 | 0.100 | constant | -3.569 | 0.251 |
| 3 | 0.136 | 0.102 | ln(shape) | -0.759 | 0.027 |

BCR – best clinical response; cECOG – collapsed Eastern Cooperative Oncology Group; ISS – international staging system; MR – Minimal Response; PR – Partial Response; PD – Progressive Disease; SD – Stable Disease; VG – Very Good Partial Response

S22 Table: Risk Equation – LoT 3 Chemotherapy Duration

| Covariate | Coefficient | Std. Err. | Covariate | Coefficient | Std. Err. |
| --- | --- | --- | --- | --- | --- |
| Age | -0.003 | 0.004 | BCR |  |  |
| Male | -0.044 | 0.078 | VG | 0.153 | 0.160 |
| cECOG |  |  | PR | 0.142 | 0.150 |
| 1 | 0.208 | 0.111 | MR | 0.058 | 0.176 |
| 2, 3 & 4 | 0.279 | 0.140 | SD | 0.151 | 0.166 |
| ISS |  |  | PD | 0.564 | 0.168 |
| 2 | -0.014 | 0.110 | constant | -4.821 | 0.320 |
| 3 | 0.155 | 0.109 | ln(shape) | -0.241 | 0.029 |

BCR – best clinical response; cECOG – collapsed Eastern Cooperative Oncology Group; ISS – international staging system; MR – Minimal Response; PR – Partial Response; PD – Progressive Disease; SD – Stable Disease; VG – Very Good Partial Response

S23 Table: Risk Equation – LoT 3 Best Clinical Response

| Covariate | Coefficient | Std. Err. | Covariate | Coefficient | Std. Err. |
| --- | --- | --- | --- | --- | --- |
| Age | 0.002 | 0.006 | BCR |  |  |
| Male | 0.088 | 0.126 | VG | 0.625 | 0.266 |
| cECOG |  |  | PR | 1.779 | 0.249 |
| 1 | 0.103 | 0.164 | MR | 1.813 | 0.282 |
| 2, 3 & 4 | 0.380 | 0.223 | SD | 3.236 | 0.295 |
| ISS |  |  | PD | 3.072 | 0.300 |
| 2 | -0.002 | 0.168 | cut1 | 0.854 | 0.464 |
| 3 | 0.083 | 0.168 | cut2 | 2.956 | 0.474 |

BCR – best clinical response; cECOG – collapsed Eastern Cooperative Oncology Group; ISS – international staging system; MR – Minimal Response; PR – Partial Response; PD – Progressive Disease; SD – Stable Disease; VG – Very Good Partial Response

S24 Table: Risk Equation – LoT 3 Treatment-free Interval

| Covariate | Coefficient | Std. Err. | Covariate | Coefficient | Std. Err. |
| --- | --- | --- | --- | --- | --- |
| Age | 0.002 | 0.004 | 3 | -0.154 | 0.129 |
| Male | 0.099 | 0.094 | cBCR |  |  |
| cECOG |  |  | PR/MR | 0.402 | 0.139 |
| 1 | 0.005 | 0.121 | SD/PD | 0.704 | 0.148 |
| 2, 3 & 4 | -0.075 | 0.149 | constant | -2.944 | 0.333 |
| ISS |  |  | ln(shape) | -0.745 | 0.035 |
| 2 | -0.030 | 0.132 |  |  |  |

cBCR – collapsed best clinical response; cECOG – collapsed Eastern Cooperative Oncology Group; ISS – international staging system; PR/MR – Partial Response/Minimal Response; SD/PD – Stable Disease/Progressive Disease

S25 Table: Risk Equation – LoT 4 Chemotherapy Duration

| Covariate | Coefficient | Std. Err. | Covariate | Coefficient | Std. Err. |
| --- | --- | --- | --- | --- | --- |
| Age | -0.010 | 0.006 | 3 | 0.138 | 0.150 |
| Male | 0.037 | 0.109 | cBCR |  |  |
| cECOG |  |  | PR/MR | 0.194 | 0.137 |
| 1 | 0.190 | 0.131 | SD/PD | 0.254 | 0.142 |
| 2, 3 & 4 | 0.107 | 0.182 | constant | -4.163 | 0.435 |
| ISS |  |  | ln(shape) | -0.229 | 0.041 |
| 2 | -0.022 | 0.144 |  |  |  |

cBCR – collapsed best clinical response; cECOG – collapsed Eastern Cooperative Oncology Group; ISS – international staging system; PR/MR – Partial Response/Minimal Response; SD/PD – Stable Disease/Progressive Disease

S26 Table: Risk Equation – LoT 4 Best Clinical Response

| Covariate | Coefficient | Std. Err. | Covariate | Coefficient | Std. Err. |
| --- | --- | --- | --- | --- | --- |
| Age | 0.017 | 0.009 | 3 | -0.069 | 0.244 |
| Male | -0.401 | 0.181 | cBCR |  |  |
| cECOG |  |  | PR/MR | 1.386 | 0.240 |
| 1 | 0.111 | 0.219 | SD/PD | 2.913 | 0.272 |
| 2, 3 & 4 | -0.102 | 0.300 | cut1 | 0.788 | 0.647 |
| ISS |  |  | cut2 | 3.048 | 0.664 |
| 2 | -0.186 | 0.240 |  |  |  |

cBCR – collapsed best clinical response; cECOG – collapsed Eastern Cooperative Oncology Group; ISS – international staging system; PR/MR – Partial Response/Minimal Response; SD/PD – Stable Disease/Progressive Disease

S27 Table: Risk Equation – LoT 4 Treatment-free Interval

| Covariate | Coefficient | Std. Err. | Covariate | Coefficient | Std. Err. |
| --- | --- | --- | --- | --- | --- |
| Age | -0.011 | 0.006 | 3 | 0.107 | 0.183 |
| Male | 0.188 | 0.137 | cBCR |  |  |
| cECOG |  |  | PR/MR | 0.345 | 0.215 |
| 1 | 0.083 | 0.162 | SD/PD | 0.662 | 0.214 |
| 2, 3 & 4 | -0.312 | 0.236 | constant | -2.388 | 0.493 |
| ISS |  |  | ln(shape) | -0.623 | 0.051 |
| 2 | -0.132 | 0.174 |  |  |  |

cBCR – collapsed best clinical response; cECOG – collapsed Eastern Cooperative Oncology Group; ISS – international staging system; PR/MR – Partial Response/Minimal Response; SD/PD – Stable Disease/Progressive Disease

S28 Table: Risk Equation – LoT 5 Chemotherapy Duration

| Covariate | Coefficient | Std. Err. | Covariate | Coefficient | Std. Err. |
| --- | --- | --- | --- | --- | --- |
| Age | 0.005 | 0.007 | 3 | 0.039 | 0.238 |
| Male | 0.187 | 0.163 | cBCR |  |  |
| cECOG |  |  | PR/MR | 0.145 | 0.218 |
| 1 | 0.206 | 0.199 | SD/PD | 0.019 | 0.215 |
| 2, 3 & 4 | 0.639 | 0.291 | constant | -4.670 | 0.601 |
| ISS |  |  | ln(shape) | -0.310 | 0.059 |
| 2 | -0.332 | 0.217 |  |  |  |

cBCR – collapsed best clinical response; cECOG – collapsed Eastern Cooperative Oncology Group; ISS – international staging system; PR/MR – Partial Response/Minimal Response; SD/PD – Stable Disease/Progressive Disease

S29 Table: Risk Equation – LoT 5 Best Clinical Response

| Covariate | Coefficient | Std. Err. | Covariate | Coefficient | Std. Err. |
| --- | --- | --- | --- | --- | --- |
| Age | -0.012 | 0.013 | 3 | -0.040 | 0.393 |
| Male | -0.288 | 0.267 | cBCR |  |  |
| cECOG |  |  | PR/MR | 1.653 | 0.381 |
| 1 | -0.159 | 0.353 | SD/PD | 3.191 | 0.422 |
| 2, 3 & 4 | 0.435 | 0.502 | cut1 | -1.128 | 0.939 |
| ISS |  |  | cut2 | 1.175 | 0.943 |
| 2 | -0.250 | 0.356 |  |  |  |

cBCR – collapsed best clinical response; cECOG – collapsed Eastern Cooperative Oncology Group; ISS – international staging system; PR/MR – Partial Response/Minimal Response; SD/PD – Stable Disease/Progressive Disease

S30 Table: Risk Equation – LoT 5 Treatment-free Interval

| Covariate | Coefficient | Std. Err. | Covariate | Coefficient | Std. Err. |
| --- | --- | --- | --- | --- | --- |
| Age | -0.017 | 0.005 | 3 | 0.096 | 0.163 |
| Male | -0.048 | 0.110 | cBCR |  |  |
| cECOG |  |  | PR/MR | 0.975 | 0.205 |
| 1 | -0.139 | 0.147 | SD/PD | 1.382 | 0.197 |
| 2, 3 & 4 | 0.030 | 0.261 | constant | -3.212 | 0.464 |
| ISS |  |  | ln(shape) | -0.269 | 0.041 |
| 2 | 0.069 | 0.124 |  |  |  |

cBCR – collapsed best clinical response; cECOG – collapsed Eastern Cooperative Oncology Group; ISS – international staging system; PR/MR – Partial Response/Minimal Response; SD/PD – Stable Disease/Progressive Disease

S31 Table: Risk Equation – LoT 6+ Chemotherapy Duration

| Covariate | Coefficient | Std. Err. | Covariate | Coefficient | Std. Err. |
| --- | --- | --- | --- | --- | --- |
| Age | -0.010 | 0.007 | 3 | 0.513 | 0.198 |
| Male | -0.014 | 0.156 | cBCR |  |  |
| cECOG |  |  | PR/MR | 0.282 | 0.245 |
| 1 | -0.371 | 0.211 | SD/PD | 0.840 | 0.236 |
| 2, 3 & 4 | -0.169 | 0.320 | constant | -4.211 | 0.630 |
| ISS |  |  | ln(shape) | -0.202 | 0.056 |
| 2 | 0.345 | 0.177 |  |  |  |

cBCR – collapsed best clinical response; cECOG – collapsed Eastern Cooperative Oncology Group; ISS – international staging system; PR/MR – Partial Response/Minimal Response; SD/PD – Stable Disease/Progressive Disease

S32 Table: Risk Equation – LoT 6+ Best Clinical Response

| Covariate | Coefficient | Std. Err. | Covariate | Coefficient | Std. Err. |
| --- | --- | --- | --- | --- | --- |
| Age | -0.002 | 0.014 | 3 | 0.024 | 0.365 |
| Male | -0.176 | 0.296 | cBCR |  |  |
| cECOG |  |  | PR/MR | 2.222 | 0.468 |
| 1 | -0.478 | 0.306 | SD/PD | 3.787 | 0.502 |
| 2, 3 & 4 | -0.040 | 0.463 | cut1 | -0.274 | 1.063 |
| ISS |  |  | cut2 | 2.635 | 1.083 |
| 2 | 0.033 | 0.345 |  |  |  |

cBCR – collapsed best clinical response; cECOG – collapsed Eastern Cooperative Oncology Group; ISS – international staging system; PR/MR – Partial Response/Minimal Response; SD/PD – Stable Disease/Progressive Disease

S33 Table: Risk Equation – LoT 6+ Treatment-free Interval

| Covariate | Coefficient | Std. Err. | Covariate | Coefficient | Std. Err. |
| --- | --- | --- | --- | --- | --- |
| Age | -0.009 | 0.009 | 3 | 0.026 | 0.269 |
| Male | -0.195 | 0.200 | cBCR |  |  |
| cECOG |  |  | PR/MR | 0.384 | 0.374 |
| 1 | 0.091 | 0.226 | SD/PD | 0.732 | 0.377 |
| 2, 3 & 4 | 0.011 | 0.335 | constant | -2.601 | 0.777 |
| ISS |  |  | ln(shape) | -0.489 | 0.074 |
| 2 | -0.142 | 0.228 |  |  |  |

cBCR – collapsed best clinical response; cECOG – collapsed Eastern Cooperative Oncology Group; ISS – international staging system; PR/MR – Partial Response/Minimal Response; SD/PD – Stable Disease/Progressive Disease
